# Use of TaqMan array card to investigate bacterial isolates in diabetic foot ulcers

**DOI:** 10.1099/jmm.0.002113

**Published:** 2026-04-17

**Authors:** Sarah Johnson-Lynn, Martin D. Curran, Candice Allen, Katherine M. Webber, Mailis Maes, Matthew Routledge, David A. Enoch, Andrew H. N. Robinson, Anthony P. Coll

**Affiliations:** 1 Department of Trauma and Orthopaedics, Cambridge University Hospitals NHS Foundation Trust, Cambridge, UK; 2 University of York, The James Cook University Hospital, Middlesbrough, UK; 3 Clinical Microbiology & Public Health Laboratory, United Kingdom Health Security Agency (UKHSA), Cambridge University Hospitals NHS, Cambridge, UK; 4 Wolfson Diabetes and Endocrine Clinic, Institute of Metabolic Science, Cambridge University Hospitals NHS Foundation Trust, Cambridge, UK

**Keywords:** diabetic foot infection (DFI), TaqMan array

## Abstract

Diabetic foot disease is a major public health problem, with an annual National Health Service (NHS) expenditure exceeding £1 billion. Infection increases the risk of major amputation fivefold. Due to the polymicrobial nature of diabetic foot infections, it is often difficult to correctly and rapidly isolate pathological organisms with conventional culture techniques and deliver appropriate narrow-spectrum antimicrobials. Rapid DNA-based technology, using multi-channel arrays, offers a quicker alternative and has previously been used effectively in other settings. We undertook a prospective cohort study of deep tissue samples taken from diabetic foot ulcers (DFUs), comparing samples processed by conventional culture and real-time PCR TaqMan array card (TAC). Fifty samples were taken from 39 patients. The ulcers were of variable chronicity prior to sampling (range: 1–113 weeks) and were sited on the heel (3), midfoot (6) and forefoot (41). TAC results were available an average of 4.3 days earlier than culture results. Seventeen samples had the same organisms detected on culture and TAC. Sixteen of these 17 had additional organisms detected by TAC. The most frequent organisms detected by TAC that were not detected by culture were staphylococci, *Enterobacter* spp., *Pseudomonas* spp. and fungi. TAC rapidly and accurately detects clinically relevant organisms from DFUs, providing earlier results than standard culture. This may enable earlier rationalization of antimicrobials and infection control interventions.

## Introduction

Diabetic foot ulcers (DFUs) are a major public health problem, with annual National Health Service (NHS) expenditure exceeding £1 billion. Infection increases the risk of major amputation fivefold [1]. Foot infection and amputation are the most feared complications of diabetes [2]. Five-year mortality in patients with DFU is 30%, increasing to 57% in those with major amputation [3]. Early infection of DFUs is frequently due to Gram-positive cocci. Polymicrobial growth with Gram-negative bacilli and fungi becomes more common later in chronic DFU [4]. There may also be synergistically acting colonies of micro-organisms protected by biofilms [5].

Identifying causative organisms rapidly and accurately, in order to deliver narrow-spectrum antimicrobials, is challenging as multidrug-resistant bacteria are increasingly being reported [6]. Current practice for clinical infection is to initiate empirical antimicrobial therapy following tissue sampling and then modify therapy after culture results. This typically takes 1–5 days before antimicrobials can be rationalized. Rapid DNA-based technology using multi-channel arrays, such as TaqMan, presents a quicker alternative and has been used in other clinical settings (e.g. respiratory infections in intensive care units [7,9]).

The aim of this study was to compare organisms detected in DFU on a TaqMan array card (TAC) with conventional culture.

## Methods

### Setting

Multidisciplinary diabetes foot clinic in Cambridge University Hospitals (CUH) NHS Trust. CUH is a tertiary referral hospital serving local and regional patients.

### Design

This was a prospective, single-centre observational cohort study, recruiting consecutive patients with a deep DFU and/or clinical suspicion of infection/osteomyelitis.

### Patients

Inclusion criteria: age ≥18 years, able to give consent. Exclusion criteria: unable to give consent, or sufficient retained sensation to prevent biopsy in clinic, pregnant or breastfeeding. As sampling was acquired using routine techniques, anticoagulated patients were included. Demographics, features of the wound, glycated haemoglobin (HbA1c) and SINBAD (Site, Ischaemia, Neuropathy, Bacterial infection, Area and Depth) score [10] were recorded. Clinical management of the patients remained at the discretion of the treating physician in line with departmental policy.

### Conventional microbiology

After wound debridement, samples for bacterial culture were obtained and sent immediately to the clinical microbiology laboratory in a sterile universal container. An aliquot or a section of the tissue was then placed in a sterile universal bottle containing saline (0.85%) and glass beads (BM0385) and vortexed to break up the tissue. Agar plates were inoculated and incubated according to the national standards [11]. Clinically relevant isolates were identified by Matrix-Assisted Laser Desorption/Ionization (MALDI) MS (Bruker MALDI Biotyper Sirius), and antimicrobial susceptibility testing was performed using disc diffusion methodology (European Committee on Antimicrobial Susceptability Testing - EUCAST).

### Development of the diabetic foot infection TAC

**Fig. 1.**
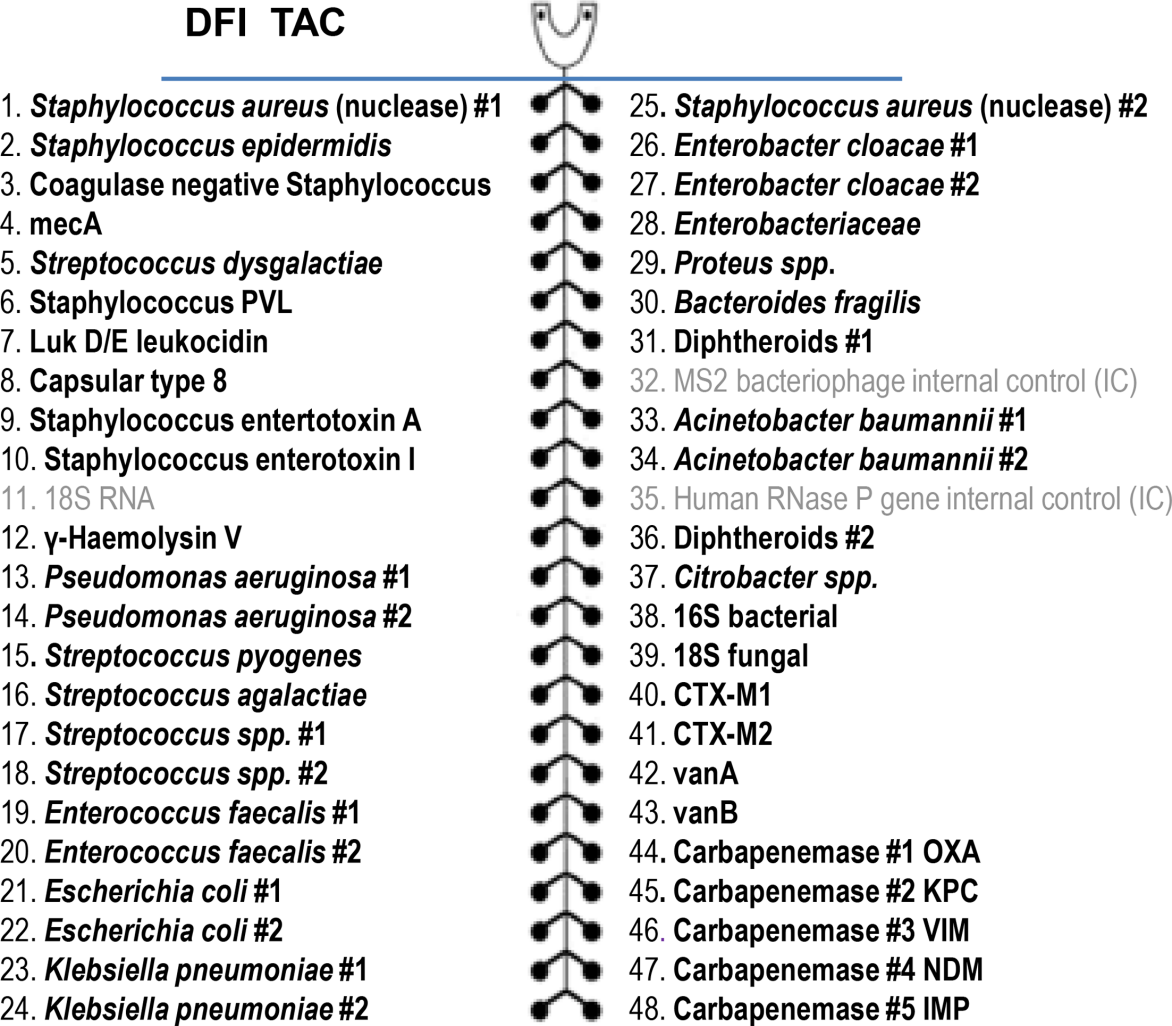
Format of TAC for investigation of diabetic foot wounds. CTX, cefotaximase.

We performed a literature review and a retrospective local audit of micro-organisms detected, to identify the most common pathogens. These pathogens were used to populate TAC, along with antimicrobial resistance genes (Fig. 1). Most real-time PCR assays used to populate the card were taken from established TACs [7,9, 12, 13]. For *Streptococcus dysgalactiae*, *Citrobacter* species, diphtheroids and *Staphylococcus aureus* virulence genes, the literature was reviewed for validated real-time PCR assays. In the absence of a published validated assay, one was designed in-house [14]. Most organisms were covered by two independent target sequences, minimizing false-positive results. Assay primer/probe sequence details are available upon request.

Specimens for diabetic foot infection (DFI) TAC analysis were processed prior to conventional microbial assessment on the remaining specimen, as outlined previously [9]. Briefly, tissue specimens (0.1–0.2 g) were first sectioned off aseptically and pulverized with a disposable scalpel. Tissue material or a 750 µl volume of homogenized material was placed into a 2 ml lysing matrix tube (mpbio.com/uk/116913050-lysing-matrix-d-cf) containing 1.4 mm zirconium beads and 750 µl of 5 M L6 guanidinium thiocyanate buffer (https://www.thistlescientific.co.uk/). Tubes were processed on a MagNa Lyser (Roche) for 1 min at 7,000 r.p.m. Tubes were centrifuged for 1 min at 13,000 r.p.m., and nucleic acid was extracted from 0.4 ml of the sample using an EZ1 Virus Mini Kit (v2.0) on an EZ1 Advanced XL (Qiagen) instrument and eluted in a 120 µl volume [12]. Extraction controls (sterile RNase-free water) were included with every extraction run.

The custom-made pre-spotted DFI TaqMan^®^ array card (TAC; Life Technologies, Carlsbad, CA, USA) was designed to include all common pathogens (Fig. 1). Each card can run up to eight samples, with 48 connected wells per sample, giving a total of 384 wells. Data were then amalgamated on the ThermoFisher QuantStudio 7 Flex platform using QuantStudio™ Real-Time PCR software v1.72. Each sample (nucleic acid) was loaded into one of the eight available slots [8]. Human RNase P and bacteriophage MS2 were used as internal controls. Reverse transcriptase real-time PCR was undertaken according to the amplification protocol: 50 °C for 5 min (reverse transcription step), 95 °C for 20 s and then 45 cycles of 95 °C for 1 s, followed by 60 °C for 20 s, with a fluorescence reading taken on the FAM (carboxyfluorescein) channel at each cycle.

DFI TACs were batch-tested on arrival against our bank of DNA extracts from a range of micro-organisms and our panel of nine synthetic control plasmids (https://www.genscript.com/) containing our target PCR sequences [9]. All wells were found to be spotted correctly, and no cross-reaction was observed.

Detection of an exponential amplification curve with a cycle threshold (Ct) value ≤38 for any gene target was reported as a positive result for that pathogen, taken as equivalent to what would be reported as scanty growth of an organism on standard culture. A Ct value of 30 was considered moderate growth, and a value of 20 as heavy growth.

## Results

Fifty samples were taken from 39 patients. Nine patients were recruited more than once due to separate episodes of clinical infection and/or new ulcers (Table 1). The mean age at enrolment was 73 years. The ulcers were of variable chronicity prior to sampling (range: 1–113 weeks; mean: 29 weeks) and were sited on the heel (3), midfoot (6) and forefoot (41). The vast majority (49/50; 98%) had a SINBAD score of ≥3, which suggests a higher risk of complications. During follow-up, 24 patients (61.5%) were admitted with complications of foot disease; there were two major amputations and four deaths (10.3% mortality rate).

**Table 1.** Demographic data

Sex	Female	8 (16%)
	Male	42 (84%)
Diabetes type	Type 2 diabetes	44 (88%)
	Type 1 diabetes	6 (12%)
Mean age (range)		73.7 (40–102)
Mean duration of ulcer/weeks (range)		29.1 (1–113)
Mean HbA1c (mmol/mol) (range)		67.2 (31–132)
SINBAD score	2	1
	3	10
	4	14
	5	20
	6	5
Type of samples	Bone	24
	Soft tissue	19
	Bone and soft tissue	7
Site of ulcer	Heel	3
	Midfoot	6
	Forefoot	41

The TAC provided results at a mean of 1.3 days. Results were available the same day for nine samples, and all but five had results the following day. Mean time to final culture results was 5.3 days (range: 2–16 days). TAC results were available a mean of 4.3 days earlier than culture results.

Seventeen samples had the same organisms detected on culture and TAC. Sixteen of these 17 (94%) had additional organisms detected by TAC. The most frequent organisms detected on the array that were not detected by culture were *Staphylococcus* spp. [including methicillin-resistant *S. aureus* (MRSA)], *Enterobacter* spp., *Streptococcus* spp. and fungi (Table 2). Extraction controls were PCR negative, ruling out the possibility that extraneous fungal/bacterial DNA was introduced via the reagents used in the nucleic acid extraction process.

**Table 2.** Organisms detected by culture and TAC (number detected by culture, number detected by PCR microarray, total number of samples positive using either technique)

	Culture No.	TAC No.	Total
**Gram-positive cocci**			
*Staphylococcus aureus*	14	17	18
Coagulase negative staphylococci	0	10*	10
*Staphylococcus capitis*	1	0	1
*Staphylococcus lugdunensis*	1	1	1
*mecA* detected	na	12	12
Methicillin resistant Staphylococcus aureus (MRSA)	5	7	7
Coagulase negative staphylococcus	0†	5	5
*Streptococcus* species	2	8	8
*Enterococcus faecalis*	7	6	8
**Gram-positive bacilli**			
*Corynebacterium* species	7	0	7
Diphtheroids	0	4	4
**Gram-negative bacilli**			
*Escherichia coli*	1	3	3
CTX-M detected	na	1	1
*Klebsiella pneumoniae*	0	2	2
*Proteus* species	3	3	4
*Citrobacter koseri*	0	2	2
*Enterobacter cloacae*	2	4	4
*Serratia marcescens*	2	2	2
*Pseudomonas aeruginosa*	8	11	11
*Stenotrophomonas maltophilia*	1	0	1
*Bacteroides fragilis*	0	1	1
*Acinetobacter baumannii*	0	1	1
*Alcaligenes faecalis*	1	0	1
*Pseudomonas* species (undifferentiated)	1	0	1
*Enterobacteriaceae* (undifferentiated)	2	2	4
Anaerobes (undifferentiated)	1	0	1
Mixed Gram-negative flora	4	3	4
**Fungi**			
Fungi (undifferentiated – 18S PCR)	0	5	5
*Candida parapsilosis*	1	0	1
**Miscellaneous**			
Mixed skin flora	15	11	16
vanA detected	na	1	1

*Coagulase negative staphylococci were most frequently reported as mixed skin flora in culture results.

†Five coagulase negative staphylococci on PCR reported as mixed skin flora on culture.

na: not applicable

CTX-M, cefotaximase.

*S. aureus* was detected in 18 samples (17 by TAC, 14 by culture). Coagulase-negative staphylococci (CNST) were detected in 11 samples (ten by TAC, two by culture were reported as *Staphylococcus capitis* and *Staphylococcus lugdunensis*, and nine were reported as mixed skin flora).

The *mecA* gene (responsible for resistance to *β*-lactamase-stable penicillins) was found in 12 samples by TAC. Of these, four detected *S. aureus* with *mecA* that appropriately grew MRSA, five detected CNST with *mecA* that appropriately grew mixed skin flora, and one detected CNST and *S. aureus* with *mecA*, which grew MRSA and mixed skin flora. Two detected *S. aureus* and CNST with *mecA* when *S. aureus* did not grow (one of these grew mixed skin flora). Cefotaximase-M1 [a common extended-spectrum *β*-lactamase (ESBL) variant] was found in one sample. Vancomycin-resistant enterococci, as indicated by the presence of vanA (resistance gene), were found in one sample (reported as mixed skin flora by culture assay). No carbapenemases were detected. Staphylococcal enterotoxin was commonly isolated (11 samples), as was haemolysin (7 samples), leucocidin (7 samples) and capsule type 8 (8 samples) by TAC.

Ten samples had no growth on culture, with six of these showing a positive reaction on TAC. Eight had a negative reaction on TAC; four of which were positive by culture.

Four samples had negative cultures and negative TAC, of whom three were from patients with ulcers present for greater than 1 year, making diagnosis of infection versus colonization more challenging. A higher proportion of samples was positive for Gram-negative organisms from ulcers present for over 6 weeks (54%) compared with those present for less than 6 weeks (43%).

## Discussion

We present data from a single centre of patients living with diabetes and foot disease. We believe that this is representative of a typical DFI service in a tertiary referral centre, and hence our observations are widely generalizable. The patients were typically elderly, with DFUs of long duration and high SINBAD scores.

The aim of this study was to compare microbiology results acquired by TAC with conventional culture.

It remains challenging to differentiate pathogenic organisms within infected DFUs rather than colonizing organisms [15]. Bacteria within chronic wounds form biofilms, increasing the difficulty of isolation and identification [4]. Bacteria detected in our population reflect those previously reported, with staphylococcal species being the most common Gram-positive isolates [16,17]. Gram-negative bacteria were commonly detected, particularly by TAC. TAC performed well in identifying organisms thought to be true pathogens, such as *S. aureus* (including MRSA), *Streptococcus* spp. and Gram-negative bacilli, whereas culture detected more enterococci and other organisms (*Corynebacterium* spp.), which may represent colonization by multiple organisms.

TAC detected *S. aureus* in 17/18 (94.4%) samples, whereas culture only detected 14 (77.8%). It detected MRSA in seven samples, whereas culture only detected five of these. It also differentiated CNST when appropriate. When linked to the improved turnaround time, this could have significant clinical and infection control implications.

PCR-based diagnostics have shown that the microbiome of chronic DFUs is more complex than suspected from culture-dependent methods. Staphylococci tend to be overestimated by culture, with the presence of anaerobes underestimated [18]. Staphylococci are the most common colonizing organisms in ulcers that are not clinically infected [19].

The duration of the ulcer has an impact on isolates identified. In our series and others, ulcers of shorter duration had predominantly Gram-positive organisms [20], while Gram-negative organisms and fungi made up a larger proportion of the microbiome with increasing duration of the ulcer [21].

TAC can provide microbiology results in a much shorter time than conventional culture, potentially allowing earlier rationalization of antimicrobials and expediting infection control interventions. A previous study demonstrated a significant cost saving with the use of molecular diagnostics to inform antimicrobial therapy [22].

Real-time PCR has been found to yield more samples positive for pathogenic staphylococcal species, including those producing toxins [23]. In our series, the virulence factors haemolysin, leucocidin D/E and enterotoxin were detected in several samples, supporting the conclusion that *Staphylococcus* in these samples was likely pathogenic. TAC also provides a Ct value for the amplification curves, facilitating a quantitative assessment of the pathogen load.

Antimicrobial-resistant organisms are often found in chronic wounds due to multiple episodes of antimicrobial therapy [20]. Resistance genes were detected in 14 of 50 samples (28%) in our series, which is lower than other series, where ESBL production and methicillin resistance were noted in 44.7% and 56.0% of bacterial isolates, respectively [24]. TACs can test directly for genes implicated in antimicrobial resistance, thereby facilitating earlier antimicrobial targeting of resistant organisms and faster implementation of infection control precautions.

The sample size of the study precludes conclusions on subgroups of patients. Further work is needed to establish the effect of this technology on earlier rationalization of antibiotics, antimicrobial stewardship and health economics. PCR-based methods can detect non-viable organisms; however, this should be weighed against the increased ability to determine between infecting and colonizing organisms. We also provide no sensitivity, specificity or positive/negative predictive value metrics. We preferred to avoid this due to the absence of a gold standard method for the presence of pathogens, but we based our results on clinical judgement. A previous study [9], however, using our TACs on patients with severe pneumonia, demonstrated a sensitivity of 92% and specificity of 97%.

TAC shows promise in detecting clinically relevant organisms from DFUs and providing earlier results than standard culture, which may enable appropriate and timely rationalization of antimicrobial therapy and faster implementation of infection control precautions. Further work is required to assess the clinical utility of this diagnostic methodology.

## Abbreviations

CNST: coagulase-negative staphylococci
Ct: cycle threshold
CUH: Cambridge University Hospitals
DFI: diabetic foot infection
DFUs: diabetic foot ulcers
ESBL: extended-spectrum *β*-lactamase
EUCAST: European Committee on Antimicrobial Susceptability Testing
MALDI: matrix-assisted laser desorption/ionization
MRSA: methicillin-resistant *Staphylococcus aureus*
NHS: National Health Service
SINBAD: site, ischaemia, neuropathy, bacterial infection, area and depth
TAC: TaqMan array card
